# Dupilumab relieves pruritus both in uremic pruritus and in atopic dermatitis with chronic kidney disease: a retrospective real-world study

**DOI:** 10.3389/fmed.2025.1627955

**Published:** 2025-09-10

**Authors:** Qian Wang, Ge Yang, Xiyuan Zhou, Xiang Zhong, Jie Liu, Lixia Zhang

**Affiliations:** ^1^Institute of Dermatology and Venereology, Sichuan Provincial People's Hospital, School of Medicine, University of Electronic Science and Technology of China, Chengdu, China; ^2^Department of Nephrology and Institute of Nephrology, Sichuan Provincial People’s Hospital, School of Medicine, University of Electronic Science and Technology of China, Sichuan Clinical Research Center for Kidney Diseases, Chengdu, China; ^3^Department of Pharmacy, Sichuan Provincial People’s Hospital, School of Medicine, University of Electronic Science and Technology of China, Chengdu, China

**Keywords:** atopic dermatitis, chronic kidney disease, dupilumab, effectiveness, pruritus

## Abstract

**Background:**

Treatment methods for pruritus in patients with chronic kidney disease (CKD) are lacking. Exploring the therapeutic potential of dupilumab in alleviating pruritus in CKD patients has good clinical value.

**Objectives:**

This retrospective study aims to analyze the effectiveness and safety of dupilumab in atopic dermatitis (AD) patients with CKD and uremic pruritus (UP) patients.

**Methods:**

Demographic and clinical data from AD patients with CKD stages 3–5 and UP patients who received dupilumab treatment were retrospectively analyzed. Improvements in pruritus were assessed via Peak Pruritus Numerical Rating Scale (PP-NRS) and 5-D itch scale (5-D IS) at weeks 2, 4, 12, and 16. Eczema Area and Severity Index (EASI) and Atopic Dermatitis Control Tool (ADCT) scores were also recorded at week 16 in AD patients with CKD. Safety during treatment was observed.

**Results:**

After dupilumab treatment, the PP-NRS and 5D-IS scores of 12 AD patients with CKD and 10 UP patients were significantly decreased. The percentages of UP patients who achieved PP-NRS ≥ 4-point improvement and 5D-IS ≤ 10-point at week 4, 12, and 16 did not significantly differ from those of AD patients with CKD (*p* > 0.05). At week 16, the skin symptoms in AD patients significantly improved (66.67% achieved EASI-75). No significant adverse effects were found.

**Conclusion:**

Dupilumab safely and effectively reduced pruritus in UP patients in the short term and achieved a comparable anti-pruritus effect to AD patients with CKD.

## 1 Introduction

Chronic pruritus is a common symptom in patients with chronic kidney disease (CKD), especially in patients with end-stage renal disease receiving hemodialysis, where the prevalence of moderate to extreme pruritus is 42% (1). Pruritus may arise mainly from uremic pruritus (UP), and CKD with primary pruritic skin diseases, such as atopic dermatitis (AD), has also been reported (2). Pruritus has a significant impact on patients, can lead to sleep disturbances, and is associated with worse mortality (3). For chronic pruritus in CKD patients, treatment is challenging. The patient’s underlying diseases, concomitant medications, and increased risk of infection limit the use of therapeutic drugs. Therefore, how to control pruritus safely and rapidly in CKD patients is an urgent clinical issue that needs to be addressed.

Dupilumab, a biologic that targets the IL-4 and IL-13 signaling pathways, has been approved for the treatment of moderate to severe AD and can result in a rapid response to itching (4). Since IL-4 and IL-13 are also important mediators of chronic pruritus, dupilumab has also been used in other chronic pruritic diseases in recent years (5–8). Compared with traditional immunosuppressive agents and JAK inhibitors, dupilumab is safer and has shown good efficacy and safety in the treatment of infants over 6 months of age and elderly patients and may be a promising treatment for complex patients with severe underlying diseases such as chronic renal insufficiency (CRI) (9–11). In recent years, only a small sample study has reported the efficacy of dupilumab in AD patients with CRI, showing that the clinical effectiveness of dupilumab in AD patients with CRI is similar to that reported in a clinical trial excluding patients with CRI (12). For the application of dupilumab in UP patients, the specific data are scarce.

Although the mechanism underlying pruritus in UP is complex, blocking critical pruritogenic mediators may be beneficial for alleviating itching. This led us to explore dupilumab for UP treatment. While a few previous case reports showed efficacy of dupilumab in UP, none compared its effectiveness to that in AD patients with CKD (13–16). In this study, we reported and compared the short-term effectiveness and safety of 16 weeks of treatment with dupilumab in 10 UP and 12 AD patients with CKD stages 3–5. This is the largest case series to date comparing UP and AD patients with CKD treated with dupilumab. This research opens up new horizons for the treatment of UP and provides further evidence for the efficacy and safety of dupilumab in controlling pruritus in CKD patients.

## 2 Methods

### 2.1 Subjects

Patients with moderate-to-severe AD and UP who completed 16 weeks of treatment with dupilumab at Sichuan provincial people’s hospital from May 2022 to March 2025 and who also had CKD stages 3–5 were included in the retrospective analysis. The stages of CKD were classified by professional nephrologists. AD was diagnosed based on the Hanifin and Rajka criteria (17). The diagnosis of UP was made by dermatologists and nephrologists based on its definition (18, 19) and the exclusion of possible comorbidities. The specific process for diagnosing UP was as follows: (1) Skin lesion review: no obvious primary lesions in patients suspected of UP, effectively excluding pruritus secondary to dermatological disorders; (2) Comorbidities review: in patients with CKD stages 3–5, pruritus should be attributed to UP in the absence of other well-defined alternative explanations. Patients were divided into an AD group and a UP group according to the two diagnoses. All patients received regular injections of dupilumab (600 mg initial dose, then 300 mg every 2 weeks) (Sanofi, France). The study conformed to the ethical standards outlined in the Declaration of Helsinki. This study was approved by the Ethics Committee of Sichuan provincial people’s hospital (NO. 2022-327), and written informed consent was obtained from the participants.

### 2.2 Data collection

Patients’ age, sex, height, weight, disease duration, underlying diseases, previous medications, adverse events during medication, and related scoring scales, such as the Peak Pruritus Numerical Rating Scale (PP-NRS), 5-D itch scale (5-D IS) (20), Dermatology Life Quality Index (DLQI), Eczema Area and Severity Index (EASI), and Atopic Dermatitis Control Tool (ADCT), were collected from patient files. The PP-NRS, 5-D IS and DLQI were assessed at baseline and 2, 4, 12, and 16 weeks after the initial dose. The proportions of patients who achieved PP-NRS ≥ 4-point improvement, 5D-IS ≤ 10-point, and DLQI ≥ 4-point improvement at each time point were compared between the AD group and the UP group. The EASI and ADCT of AD patients were recorded at baseline and 16 weeks after the initial dose. Data collection was performed independently by two experienced dermatologists.

### 2.3 Statistical analyses

All the data were analyzed via SPSS version 26.0 (IBM, New York, NY, USA). Descriptive analysis was employed to examine demographic data and clinical characteristics. Continuous variables are presented as the means ± standard deviations, and categorical variables are described as percentages. Comparisons of continuous variables between two groups were conducted via independent samples *t* test or paired samples *t* test or Mann–Whitney U test. Fisher’s exact test was performed to analyze categorical variables. Given the small sample size (n < 30), no correction for multiple comparisons was implemented to avoid overly conservative statistical inferences, which could increase the risk of Type II errors. All tests were two-sided, *p* < 0.05 was considered statistically significant.

## 3 Results

### 3.1 Patient characteristics

A total of 12 AD patients and 10 UP patients were included, all of whom had comorbid CKD stages 3–5 and were receiving a standard dose of dupilumab for 16 weeks, 14 of whom were hemodialysis patients (6 in AD group and 8 in UP group). The primary causes of CKD include: diabetes mellitus (7 cases), hypertension (5 cases), ANCA-associated vasculitides (2 cases), with an additional 8 patients unable to provide relevant information. In the AD group, 8 males and 4 females aged 39–91 years (70.75 ± 14.95) were included. In the UP group, 8 males and 2 females were aged 55–84 years (72.40 ± 10.10). There were no significant differences in gender and age between the two groups. Some comorbidities like allergic rhinitis, asthma, hypertension or cardiovascular disease, diabetes and so on were observed (Table 1). Medications for these comorbidities while the patients were on dupilumab were shown in Supplementary Table 1. All patients had previously used oral antihistamines, emollients and topical glucocorticoids. In addition, some patients used other systemic medications, like gabapentin. Prior systemic therapeutic medications were shown in Supplementary Table 2. They discontinued other medications for itching after receiving dupilumab. All patients had significant pruritus at baseline and there was no statistically significant difference in the intensity of itching between the two groups (Table 1). The clinical and demographic data of the patients are presented in Table 1.

**Table 1:** Clinical and demographic data of AD and UP patients concomitant with CKD stages 3–5 at baseline.

Characteristics	AD with CKD (*n* = 12)	UP (*n* = 10)	*p* value
Sex, male (%)	8 (66.67)	8 (80.00)	0.417
Age, year	70.75 ± 14.95	72.40 ± 10.10	0.770
BMI	22.94 ± 2.71	22.31 ± 2.56	0.584
Duration, years	4.13 ± 7.18	2.65 ± 2.87	0.923
Clinical scores			
PP-NRS	8.33 ± 0.98	7.40 ± 1.43	0.086
5D-IS	16.50 ± 2.02	15.40 ± 1.78	0.195
DLQI	12.58 ± 3.50	11.50 ± 5.66	0.123
EASI	24.00 ± 7.13	/	/
ADCT	20.25 ± 3.17	/	/
Comorbidities (%)			
Allergic rhinitis	5 (41.67)	4 (40.00)	/
Asthma	2 (16.67)	1(10.00)	/
Hypertension or cardiovascular system	8 (66.67)	7 (70.00)	/
Diabetes	7 (58.33)	4 (40.00)	/
Hyperuricemia	2 (16.67)	5 (50.00)	/
COPD	3 (25.00)	3 (30.00)	/
Ischemic stroke	1 (8.33)	1 (10.00)	/
Thyroid disease	2 (16.67)	/	/

AD, atopic dermatitis; CKD, chronic kidney disease; UP, uremic pruritus; BMI, body mass index; PP-NRS, peak pruritus numerical rating scale; 5-D IS, 5-D itch scale; DLQI, Dermatology Life Quality Index; EASI, Eczema Area and Severity Index; ADCT, Atopic Dermatitis Control Tool; COPD, chronic obstructive pulmonary disease.

### 3.2 Effectiveness assessments

#### 3.2.1 PP-NRS

**Figure 1:**
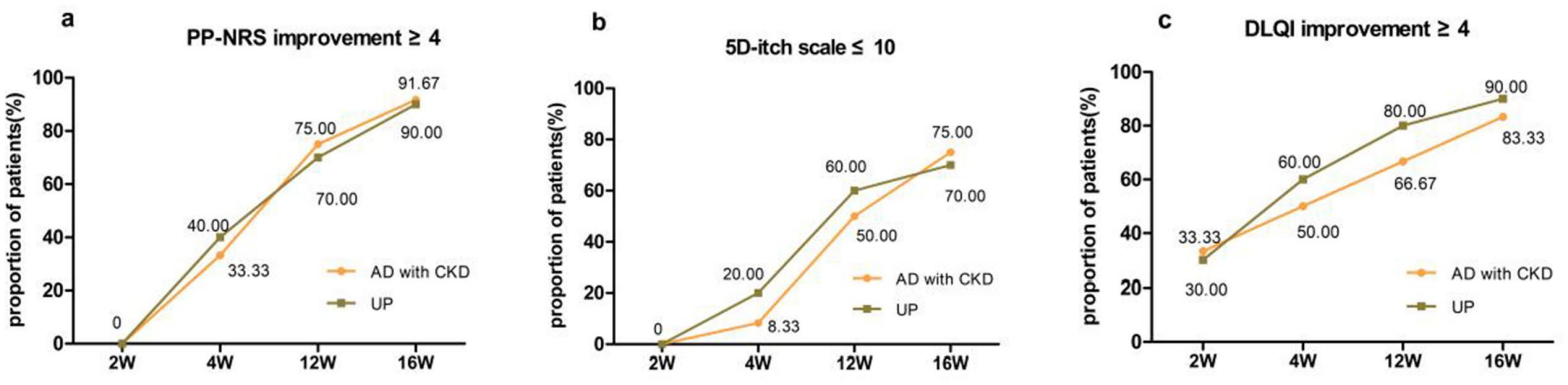
Percentages of AD patients with CKD and UP patients who achieved **(a)** PP-NRS improvement ≥ 4 points **(b)** 5D-IS ≤ 10 points, and **(c)** DLQI improvement ≥ 4 points at week 2, week 4, week 12, and week 16 after dupilumab treatment.

The PP-NRS scores in the AD group and UP group were significantly lower at 2, 4, 12, and 16 weeks after dupilumab treatment than at baseline (*p* < 0.05) (Table 2). The proportions of patients who achieved PP-NRS ≥ 4-point improvement at week 2, 4, 12, and 16 in the AD group were 0, 33.30, 75.00, and 91.67%, respectively. In the UP group, the corresponding percentages were 0, 40.00, 70.00, and 90.00%, respectively. At week 4, 12, and 16, the proportion of patients who achieved PP-NRS ≥ 4-point improvement in the UP group was not significantly different from that in the AD group (*p* = 0.546, 0.583, 0.714) (Figure 1a).

**Table 2:** Improvements in pruritus and quality of life in AD patients with CKD and UP patients after dupilumab treatment.

Group	Time point	PP-NRS		5D-IS		DLQI	
		mean ± SD	*p*	mean ± SD	*p*	mean ± SD	*p*
AD with CKD	Baseline	8.33 ± 0.98		16.50 ± 2.02		12.58 ± 3.50	
	Week 2	6.08 ± 1.31	0.000^*^	13.92 ± 1.56	0.000^*^	9.75 ± 3.14	0.000
	Week 4	5.08 ± 0.79	0.000^*^	12.50 ± 1.38	0.000^*^	8.33 ± 2.71	0.000^*^
	Week 12	3.58 ± 1.16	0.000^*^	10.58 ± 1.38	0.000^*^	6.75 ± 3.14	0.000^*^
	Week 16	2.83 ± 1.27	0.000^*^	9.42 ± 1.78	0.000^*^	4.83 ± 2.48	0.000^*^
UP	Baseline	7.40 ± 1.43		15.40 ± 1.78		11.50 ± 5.66	
	Week 2	5.70 ± 1.57	0.000^*^	13.88 ± 2.10	0.011^*^	8.60 ± 5.08	0.005^*^
	Week 4	4.40 ± 1.71	0.000^*^	11.90 ± 2.18	0.000^*^	7.40 ± 4.35	0.005^*^
	Week 12	3.20 ± 1.48	0.000^*^	10.20 ± 1.75	0.000^*^	5.50 ± 3.78	0.005^*^
	Week 16	2.30 ± 1.70	0.000^*^	8.90 ± 2.18	0.000^*^	4.40 ± 3.92	0.005^*^

*p < 0.05 compared with baseline. AD, atopic dermatitis; CKD, chronic kidney disease; UP, uremic pruritus; PP-NRS, peak pruritus numerical rating scale; 5-D IS, 5-D itch scale; DLQI, dermatology life quality index.

#### 3.2.2 5-D IS

The 5D-IS scores were significantly lower at 2, 4, 12, and 16 weeks in the AD group and UP group after dupilumab treatment than at baseline (*p* < 0.05) (Table 2). The proportions of patients who reached 5D-IS ≤ 10-point at week 2, 4, 12, and 16 in the AD group were 0, 8.33, 50.00, and 75.00%, respectively. The corresponding proportions of patients in the UP group were 0, 20.00, 60.00, and 70.00%, respectively. At week 4, 12, and 16, the proportion of patients who achieved 5D-IS ≤ 10 in the UP group was not significantly different from that in the AD group (*p* = 0.429, 0.485, 0.583) (Figure 1b).

#### 3.2.3 DLQI

After dupilumab treatment, the DLQI scores at week 2, 4, 12, and 16 in the AD and UP groups were significantly lower than the corresponding baseline scores (*p* < 0.05) (Table 2). The proportions of patients who achieved DLQI ≥ 4-point improvement at week 2, 4, 12, and 16 in the AD group were 33.33, 50.00, 66.67, and 83.33%, respectively. The corresponding percentages of patients in the UP group were 30.00, 60.00, 80.00, and 90.00%, respectively. At weeks 4, 12, and 16, the proportion of patients who achieved DLQI ≥ 4-point improvement was not significantly different between the UP group and the AD group (*p* = 0.485, 0.417, 0.571) (Figure 1c).

#### 3.2.4 EASI and ADCT in AD patients with CKD

After dupilumab treatment at week 16, the EASI score of 12 AD patients with CKD decreased significantly from baseline (24.00 ± 7.13 vs. 6.08 ± 5.03) (t = 14.042, *p* = 0.000). The EASI-50, EASI-75, and EASI-90 responses were 91.67, 66.67, and 16.67%, respectively. The ADCT score also decreased from 20.25 ± 3.17 to 7.25 ± 2.96 (t = 13.100, p = 0.000), with 50.00% of patients achieving ADCT < 7.

#### 3.2.5 Safety

Two patients developed mild conjunctivitis during the treatment, which resolved after using tobramycin and dexamethasone eye ointment and hyaluronic acid sodium eye drops. No patients developed injection site reactions, facial or neck erythema, or other complications. No patients reported deterioration of renal function or changes in the hemodialysis regimen. No patients stopped using dupilumab as a result of side effects.

## 4 Discussion

This study retrospectively analyzed the short-term effectiveness of dupilumab in treating pruritus in CKD patients. This supported that dupilumab had a rapid onset of action and achieved the intended goal in a short period of time in AD patients with CKD, which is consistent with previous reports (12). Moreover, this study revealed the advantages of dupilumab in overcoming pruritus at an early stage in UP patients, and the anti-itching effects of dupilumab on UP patients and AD patients with CKD were not significantly different.

Uremic pruritus (UP), also known as chronic kidney disease-associated pruritus (CKD-aP), is a common symptom in CKD patients, with a high disease burden and is difficult to treat. UP is more common among hemodialysis patients, but pruritus may occur in both dialysis and nondialysis CKD patients (21). The common comorbidities of UP are hypertension and diabetes (22), and our findings are consistent with these findings. The presence of comorbidities such as diabetes may make itching more difficult to control. Additionally, some CKD patients may have comorbid primary pruritic dermatosis, which may exacerbate itching in these patients. Impairment of the normal reaction of the innate and adaptive immune systems in CKD predisposes patients to an increased risk of infection (23), and skin excoriation due to scratching may increase the risk. Chronic pruritus also has a significant negative impact on social psychology and quality of life for patients. Thus, rapid control of pruritus in the short term is very important in CKD patients, but in practice, it is not easy. This study suggests that dupilumab may be a fast-acting and safe therapy for pruritus in CKD patients.

Dupilumab is a fully human IgG4 monoclonal antibody directed against the interleukin-4 receptor subunit *α* (IL-4Rα) of the IL-4 and IL-13 receptors and has been approved in some countries and regions for the treatment of patients with moderate to severe AD. CHRONOS, OLE and a series of real-world studies have confirmed its efficacy and safety (24–27). Dupilumab has been reported to be a promising drug for AD patients with cancer, HIV infection, liver disease, kidney disease, and organ transplantation (28–30). A retrospective study that included 18 AD patients with CKD revealed that dupilumab successfully improved pruritus and clinical scores (12), which is the largest number of cases of AD with CKD that have been reported with dupilumab treatment. This study revealed that the percentage of PP-NRS ≥ 4-point improvement at week 16 was much greater than that reported in other clinical trials, suggesting that dupilumab may be beneficial for UP (12). In our study, 12 AD patients with CKD showed significant improvement in skin lesions after treatment with dupilumab, with 66.67% of patients achieving EASI-75 scores at week 16. The percentages of AD patients who achieved PP-NRS ≥ 4-point improvement at weeks 4, 12, and 16 were slightly lower but close to those reported in previous studies (33.33% vs. 50%, 75.00% vs. 77.8%, 91.67% vs. 93.7%) (12), supporting the notion that dupilumab has a positive effect on UP. Given the very small number of cases reported on the use of dupilumab for UP (13–16) and the lack of relevant experience in clinical application, we simultaneously observed the effectiveness of dupilumab in 10 patients with UP and compared it with the 12 AD patients with CKD. Our study revealed that dupilumab relieved pruritus in UP patients at an early stage, with 40% achieving PP-NRS ≥ 4-point improvement and 20% achieving a 5D-IS score ≤ 10 points at week 4 and 90 and 70% achieving this level of improvement at week 16, respectively, as well as a significant improvement in patients’ quality of life. This degree of improvement in pruritus and quality of life was not significantly different from that in AD patients with CKD, suggesting that dupilumab has potential efficacy in alleviating pruritus symptoms in patients with UP. In addition, the study included 14 hemodialysis patients, and hemodialysis did not affect the efficacy of dupilumab. It is noteworthy that all patients in this study discontinued other systemic medications for itching after initiating dupilumab therapy, indicating that dupilumab may reduce CKD patients’ reliance on other antipruritic agents (such as antihistamines and gabapentin). This alleviates the burden of systemic drugs on renal and hepatic function, thus further enhancing treatment safety.

The underlying mechanisms involved in relieving uremic pruritus by dupilumab may include the following: (1) Blocking the IL-4Rα signaling pathway: animal experiments have shown that ablation of IL-4Rα inhibits the development of chronic pruritus in mice (31), and dupilumab may block the signaling of pruritus by blocking the IL-4Rα signaling pathway. (2) Reducing the expression of IL-31 by inhibiting the IL-4/IL-13 signaling pathway (32): Dupilumab may alleviate pruritus in UP patients by reducing IL-31 levels, as L-31 levels are higher in UP patients than in nonpruritic hemodialysis patients (33). (3) Reduction in *S. aureus* colonization: *S. aureus* directly activates pruritus-sensing neurons and evokes spontaneous pruritus through the *S. aureus* serine protease V8 (34). Pruritic hemodialysis patients have higher relative *S. aureus* counts on their skin than nonpruritic hemodialysis patients do (35), whereas dupilumab increases microbial diversity and reduces *S. aureus* colonization (36). However, because UP has numerous potential pruritogens, such as toxins, Th1 cells, and peripheral neuropathy (18), and because dupilumab only targets IL-4Rα, its antipruritic effect on UP remains to be observed, and the sample size needs to be expanded.

Dupilumab has a favorable safety profile. Narla et al. reviewed the adverse reactions reported in the literature for dupilumab, including paradoxical head and neck erythema, ocular complications, arthritis, alopecia, and psoriasiform eruptions (37), but most of them were mild, and the proportion of patients who discontinued treatment due to adverse reactions was low. None of the patients in our study experienced significant adverse effects during follow-up, suggesting that dupilumab was well tolerated in patients with CKD.

This study has inevitable limitations. First, this study only assessed the effectiveness for 16 weeks, and a longer treatment period is necessary. Second, this was a single-center study, which limits the generalizability of the findings. Third, since this was a retrospective study with a small sample size, inherent potential biases, such as selection bias, were inevitable. Fourth, sample size calculation was not performed. However, our study may provide a more clinically relevant real-life scenario. Of course, the 16-week follow-up period is insufficient to assess long-term efficacy or late adverse effects. Therefore, larger multicenter prospective randomized controlled studies with longer follow-up period are necessary to further evaluate the long-term efficacy, safety, and potential risks of dupilumab in UP and CKD combined with other primary pruritic dermatoses.

## 5 Conclusion

This study demonstrated that dupilumab had potential efficacy in short-term relief of clinical signs and symptoms in CKD stage 3–5 patients, including those with UP and AD. The improvement in pruritus and quality of life in UP patients by dupilumab treatment within 16 weeks is consistent with its effect on AD patients with CKD, and it has good safety. The results of this study provide some guidance for the appropriate use of dupilumab in the CKD population.

## Data Availability

The original contributions presented in the study are included in the article/Supplementary material, further inquiries can be directed to the corresponding authors.
