# Supplementary material for: Dupilumab relieves pruritus both in uremic pruritus and in atopic dermatitis with chronic kidney disease: a retrospective real-world study
Source: Front Med (Lausanne). 2025 Sep 10;12:1627955. doi: 10.3389/fmed.2025.1627955 (PMC12457180; doi:10.3389/fmed.2025.1627955)
Supplement: Supplementary file 1 [file Table_1.DOCX]

**Supplementary Table 1.** Concomitant therapies while the patients were on dupilumab

| **Concomitant therapies** | **AD with CKD (n=12)** | **UP(n=10)** |
| --- | --- | --- |
| Insulin | 4 (33.33) | 2 (20.00) |
| Erythropoietin | 5 (41.67) | 5 (50.00) |
| Alishantins | / | 1 (10.00) |
| Clopidogrel Bisulfate | 3 (25.00) | 2 (20.00) |
| Nifedipine | 3 (25.00) | 6 (60.00) |
| Amlodipine | 2 (16.67) | / |
| Diltiazem | / | 1 (10.00) |
| Valsartan | 1 (8.33) | 3 (30.00) |
| Arotinolol | 1 (8,33) | 1 (10.00) |
| Digoxin | 2 (16,67) | / |
| Rosuvastatin | / | 2 (20.00) |
| Atorvastatin | 3 (25.00) | 1 (10.00) |
| Compound α-Ketoacid Tablets | 2 (16.67) | 5 (50.00) |
| Sodium Zirconium Cyclosilicate | / | 4 (40.00) |
| Sevelamer | 2 (16.67) | 3 (30.00) |
| Empagliflozin | 1 (8.33) | 1 (10.00) |
| Dapagliflozin | / | 1 (10.00) |
| Linagliptin | 1 (8.33) | 1 (10.00) |
| Repaglinide | 1 (8.33) | 1 (10.00) |
| Finerenone | / | 1 (10.00) |
| L-Carnitine | / | 4 (40.00) |
| Roxadustat‌ | 1 (8.33) | 3 (30.00) |
| Febuxostat | 1 (8.33) | 4 (40.00) |
| Metoprolol | 2 (16.67) | 2 (20.00) |
| Iron Polysaccharide Complex | / | 2 (20.00) |
| Trimetazidine | 1 (8.33) | 1 (10.00) |
| Piperazine Ferulate | 1 (8.33) | 1 (10.00) |
| Terazosin | 1 (8.33) | 2 (20.00) |
| Calciferol | 2 (16.67) | 2 (20.00) |

AD, atopic dermatitis; CKD, chronic kidney disease; UP, uremic pruritus.

**Supplementary Table 2.** Prior systemic treatments for AD and UP patients

| **Previous systemic treatment** | **AD with CKD (n=12)** | **UP(n=10)** |
| --- | --- | --- |
| Systemic antihistamines | 12 (100.00) | 10 (100.00) |
| Chinese traditional medicine | 5 (51.67) | 2 (20.00) |
| Systemic glucocorticoids | 4 (33.33) | / |
| Gabapentin/Pregabalin | 2 (16.67) | 4 (40.00) |
| Upadacitinib | 1 (8.33) | / |

AD, atopic dermatitis; CKD, chronic kidney disease; UP, uremic pruritus.
